# May *Staphylococcus* *lugdunensis* Be an Etiological Factor of Chronic Maxillary Sinuses Infection?

**DOI:** 10.3390/ijms23126450

**Published:** 2022-06-09

**Authors:** Maja Kosecka-Strojek, Mariola Wolska-Gębarzewska, Adrianna Podbielska-Kubera, Alfred Samet, Beata Krawczyk, Jacek Międzobrodzki, Michał Michalik

**Affiliations:** 1 Department of Microbiology, Faculty of Biochemistry, Biophysics and Biotechnology, Jagiellonian University in Krakow, Gronostajowa 7, 30-387 Krakow, Poland; mariola.wolska@doctoral.uj.edu.pl (M.W.-G.); jacek.miedzobrodzki@uj.edu.pl (J.M.); 2 MML Centre, Bagno 2, 00-112 Warsaw, Poland; adrianna.podbielska@mml.com.pl (A.P.-K.); dr.alfredsamet@gmail.com (A.S.); michal.michalik@mml.com.pl (M.M.); 3 Department of Molecular Biotechnology and Microbiology, Faculty of Chemistry, Gdansk University of Technology, Gabriela Narutowicza 11/12, 80-233 Gdansk, Poland; beata.krawczyk@pg.edu.pl

**Keywords:** coagulase-negative staphylococci, MLST, virulence factors, biofilm, laryngological infections

## Abstract

*Staphylococcus* *lugdunensis* is an opportunistic pathogen found in the healthy human skin microbiome bacterial community that is able to cause infections of diverse localization, manifestation, and course, including laryngological infections, such as necrotizing sinusitis. Chronic maxillary sinusitis is a disease present in up to one third of European and American populations, and its etiology is not fully described. Within this study, we aimed to characterize 18 *S. lugdunensis* strains recovered from maxillary sinuses and evaluate them as etiological agents of chronic disease. We performed MLST analysis, the complex analysis of both phenotypic and genetic virulence factors, antibiotic susceptibility profiles, and biofilm formation assay for the detection of biofilm-associated genes. Altogether, *S. lugdunensis* strains were clustered into eight different STs, and we demonstrated several virulence factors associated with the chronic disease. All tested strains were able to produce biofilm in vitro with numerous strains with a very strong ability, and overall, they were mostly susceptible to antibiotics, although we found resistance to fosfomycin, erythromycin, and clindamycin in several strains. We believe that further in-depth analysis of *S. lugdunensis* strains from different niches, including the nasal one, should be performed in the future in order to reduce infection rate and broaden the knowledge about this opportunistic pathogen that is gaining attention.

## 1. Introduction

*Staphylococcus lugdunensis* has been known since the late 1980s as a component of the human skin microbiome but also as a potential dangerous pathogen [1]. It is mainly associated with the lower parts of the human body but can also be found in the nasal cavity, causing a wide range of infections, including severe ones with high mortality rates [2,3,4]. Moreover, *S. lugdunensis* infections may be underestimated since a positive result, as with *S. aureus*, may be identified with routinely used agglutination tests [5]. The invasive infections are also detected at an insufficient level since coagulase negative staphylococci (CoNS) are often discarded as contaminants. Since the matrix-assisted laser desorption ionization–time of flight mass spectrometry (MALDI TOF MS) method was introduced into routine microbiological practice, *S. lugdunensis* infections are detected at higher levels [6,7]. Still, chronic infections, such as chronic maxillary sinusitis, are rarely related to *S. lugdunensis* as an etiological factor [8]. However, it is known that CoNS are responsible for laryngological infections, and there are reports describing *S. lugdunensis* as responsible for acute necrotizing sinusitis in hospitalized patients with metastatic prostate adenocarcinoma, rhinosinusitis, nasal polyps, and osteomyelitis, which can lead to laryngological implications in terms of significant effects on the head or face bones [8].

Acute rhinosinusitis (ARS) appears the most frequently in the course of a viral cold disease, whereas chronic rhinosinusitis (CRS) is commonly associated with CoNS [9]. Chronic rhinosinusitis is one of the most common chronic disorders, present in up to 28% of European or American populations, and its mechanisms are still under careful molecular analysis [10]. Bacterial infection plays an important role in CRS as either a causative or exacerbating factor. The microbiological analysis of samples from CRS patients revealed that mainly CoNS were identified, followed by *S. aureus* and gram-negative rods, including *Pseudomonas aeruginosa*, *Stenotrophomonas maltophilia*, *Escherichia coli*, and *Serratia marcescens* [11,12,13,14]. It was shown that 87% of CRS cases were due to CoNS, with the most frequently isolated being *S. epidermidis*, *S. lugdunensis*, *S. capitis*, *Staphylococcus saprophyticus*, *S. haemolyticus*, and *Staphylococcus saccharolyticus* [8]. Based on previous studies, it was depicted that the pathologic ability of CoNS such as *S. epidermidis* depends on genes associated with biofilm formation both in CRS but also in all other infections [10,15]. However, knowledge of the importance of bacteria and microbial biofilms in the etiology of CRS is still incomplete. Moreover, it is still not unambiguously shown that the severity of CRS diseases is associated with CoNS solely; further complex analysis based on isolates recovered from patients with CRS are needed. The frequency of CoNS isolation at the infection site and the molecular typing of CoNS to determine the clonality of the isolates is crucial in determining the real etiological factor.

In the case of any chronic infection, not only the detection of biofilm formation but also other virulence factors may play an important role. *S. lugdunensis* is able to produce several virulence factors such as (i) toxins: haemolysins *hlb* and *hlIII*, phenol-soluble modulins—synergistic hemolysins slush; (ii) cell wall modifications: *dltA*—D-Alanylation of teichoic acids and *mprF*—multiple peptide resistance factor; (iii) capsular polysaccharide Cap; (iv) adhesins/biofilm synthesis: polysaccharide intercellular adhesin *ica*, *atlL*, *eno*, *ebpS*, *fbl*—fibrinogen binding protein of *S. lugdunensis*, *vwbl*—von Willebrand factor binding protein of *S. lugdunenesis*; (v) proteolytic activity: lugdulysin—metalloprotease with homology to the *ShpI* protease of *S. hyicus* but not to *S. aureus* serine or cysteine proteases; and (vi) iron surface determinants: iron-regulated surface determinant Isd proteins (*isdC)*, iron-regulated ECF heme transporter Lha [4].

In the case of *S. lugdunensis* species, there is one more very interesting feature, namely that it encodes two nonribosomal peptide synthesis systems. One of them is responsible for the synthesis of lugdunin—the new class of cyclic peptide antibiotic with an important antimicrobial and immunomodulatory activity [16]. Lugdunin has antimicrobial activity towards *S. aureus* via the enhancement of the innate immune response. It was also shown that individuals who are colonized with *S. lugdunensis* are less likely colonized with *S. aureus*, and the suppression of *S. aureus* growth in healthy skin and nares was observed [17,18,19]. In the nasal cavity, it may also depend on the antibiotic action and nutritional competition of these two species. Moreover, it is also considered that predominant niches of *S. lugdunensis* colonization are skin areas and not the nasal cavity, and *S. aureus* is rarely found on the skin so it is possible that *S. lugdunensis* helps to exclude *S. aureus* from healthy skin [20].

The aim of this study was to characterize *S. lugdunensis* isolates recovered from chronic maxillary sinusitis. We performed the multilocus sequence typing, complex analysis of both phenotypic and genetic virulence factors, antibiotic susceptibility profiles, and biofilm formation assay with detection of biofilm-associated genes in order to compare those results with laryngological patients’ characteristics.

## 2. Results

### 2.1. Isolates and Patients’ Characteristics

All 18 *S. lugdunensis* isolates were recovered from maxillary sinuses of patients with a chronic disease between 2017 and 2020. The patients were treated in MML Centre due to chronic sinusitis, but some of them also had nasal polyps and snoring problems (Table 1). All patients were male and adult, aged from 26 to 59. Almost all patients had undergone functional endoscopic sinus surgery (FESS). Only two patients did not have any accompanying flora isolated at the time of *S. lugdunensis* isolation. Altogether, six patients had both *S. lugdunensis* and *S. aureus* in maxillary sinuses. Two of them before *S. lugdunensis* infection, one at the *S. lugdunensis* isolation, and three were infected with *S. aureus* after *S. lugdunensis* isolation.

### 2.2. Species Identification and Antibiotic Susceptibility

**Figure 1:**
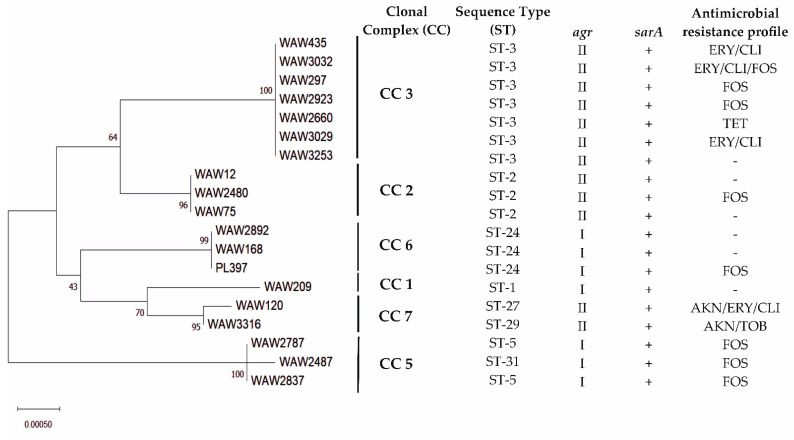
Sequence types (STs), *agr* types, *sarA* gene distribution, and antibiotic resistance profiles of *Staphylococcus lugdunensis* strains. A phylogenetic tree was constructed based on *S. lugdunensis* strains MLST results with Neighbor-Joining method in MEGA X (v. 10.2.4) [21]. The percentage of replicate trees in which the associated taxa clustered together in the bootstrap test (1000 replicates) are shown next to the branches. To identify clonal complexes (CC), BURST analysis was conducted in Bacterial Isolate Genome Sequence Database (BIGSdb) curated by Institut Pasteur, Paris, France. Antibiotics, AKN: amikacin; ERY: erythromycin; CLI: clindamycin; FOS: fosfomycin; TET: tetracycline; TOB: tobramycin; The hyphen mark means susceptibility to all antibiotics tested in the study.

All isolates were identified unambiguously as *S. lugdunensis* by 16S rRNA and *rpoB* genes sequencing. Generally, *S. lugdunensis* strains were mostly susceptible for tested antibiotics. Eight strains were resistant to fosfomycin with a MIC range of 48 to 256 mg/L (including reference strain PL397). A total of four strains were resistant to erythromycin, and all also showed resistance to clindamycin. Single strains showed resistance for amikacin, tetracycline, and tobramycin (Figure 1).

### 2.3. Multilocus Sequence Types

Based on the *S. lugdunensis* MLST database, all strains were assigned to six CCs (clonal complexes) including eight STs. The largest cluster was ST3, which grouped seven strains. Next, three strains were assigned to ST2, two to ST24 (together with reference strain PL397), and two to ST5. The remaining four strains were assigned to ST1, ST27, ST29, and ST31 (Figure 1).

### 2.4. Virulence Factors Distribution

**Figure 2:**
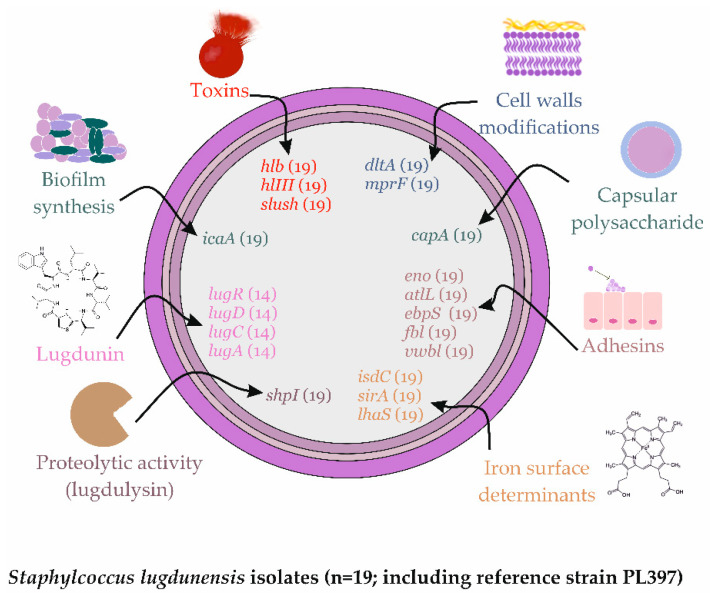
Virulence factors carried by *S. lugdunensis* strains.

All *S. lugdunensis* strains showed beta hemolysis in vitro and had *hlb*, *hlIII*, and SLUSH genes (including reference strain PL397). Five strains showed very high (incl. PL397) and nine strains high proteolysis, and five strains did not show proteolysis in vitro. All strains had a *sarA* gene and most strains had *agr* type II, with seven strains demonstrating type I (incl. PL397). Altogether, all strains possessed virulence genes associated with: cell wall modifications (*dltA*, *mprF*); capsular polysaccharide (*capA*); adhesion (*eno*, *atlL*, *ebpS*, *fbl*, *vwbl)*; proteolytic activity (*shpI*); and iron acquisition (*isdC*, *sirA* and *lhaS*). A total of 14 strains produced lugdunin in vitro and possessed *lugRDCA* genes (incl. PL397), whereas the remaining five strains did not (Figure 2).

### 2.5. Biofilm Formation

**Figure 3:**
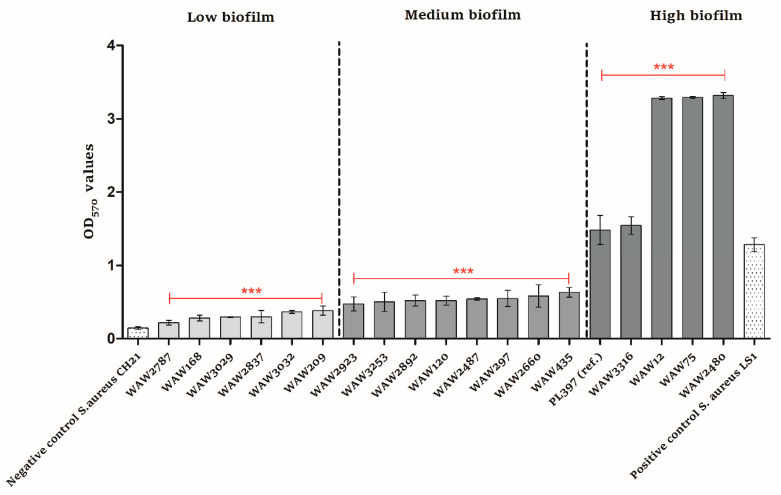
Biofilm formation in vitro of *Staphylococcus lugdunensis* strains. Each bar represents mean ± standard deviation. Dotted bars: negative and positive controls; light gray bars: low biofilm formation; medium gray bars: medium biofilm formation; dark gray bars: high biofilm formation. Experiments were performed in four repetitions. Normality was checked with Shapiro–Wilk test. Biofilm formation experimental data were analyzed using ANOVA test followed by the Bonferroni’s multiple comparison test to evaluate the difference between groups (*** *p* < 0.0001).

Compared to the negative and positive controls, all *S. lugdunensis* strains produced biofilm in vitro (Figure 3). Six strains did so at a low level (OD_570_ values from 0.15 to 0.38), eight strains at a medium level (OD_570_ values from 0.39 to 1.27), and four strains at a high level (OD_570_ values above 1.28). The reference *S. lugdunensis* strain (PL397) produced biofilm at a high level. Moreover, all strains possessed the *icaA* gene (Figure 2).

## 3. Discussion

Four decades after the first description of *Staphylococcus lugdunensis*, much more is known about its pathogenicity and virulence potential. Based on current research, this species should not be discarded as a harmless contaminant from clinical samples and should be considered as a dangerous opportunistic pathogen [4]. *S. lugdunensis* represents one of the most aggressive CoNS species, causing severe infections, especially infective endocarditis and soft tissue infections [22,23]. This is due to the fact that *S. lugdunensis* possesses a number of virulence factors associated mainly with toxic/haemolytic potential, acquisition of heme, cell wall modifications, and adhesins. Although, the number of virulence factors is not as high as that of *S. aureus* [24]. It is also known that *S. aureus* and *S. lugdunensis* can share the niche; this becomes even more interesting in light of the fact that *S. lugdunensis* produces the antibiotic lugdunin, which kills *S. aureus*. However, there are also reports where individuals colonized with *S. aureus* did not have *S. lugdunensis* in their nose [16].

Chronic maxillary sinusitis is the term generally used to describe nasal congestion or discharge that persists for 8 to 12 weeks. Chronic disease rarely causes symptoms of pain except during acute exacerbations [9]. Chronic sinusitis disease is usually bacterial rather than viral, and the most commonly isolated are Gram-positive cocci. Between them, *S. lugdunensis* was also observed, but there are not much data regarding the actual recognition of this species as an etiological factor of chronic maxillary sinuses disease [8]. Within this study, we aimed to characterize 18 *S. lugdunensis* strains recovered from the maxillary sinuses of patients with a chronic disease between 2017 and 2020 treated in MML Centre. First of all, we performed MLST analysis in order to determine the strains clonality. Altogether, the strains were assigned to six CCs including eight different STs, revealing high genetic diversity. The most common were ST3 and ST2, as also demonstrated in other studies in Europe [25]. Based on recent research, *S. lugdunensis* displays a closed pan genome, and all published genomes are very similar, with similar genes set among the strains [26]. Even though this was not expected, it can explain the very well conserved antimicrobial susceptibility of *S. lugdunensis* that rarely acquires resistance genes [27]. So, as expected, this study also demonstrated that all *S. lugdunensis* strains were similar based on genes’ distribution. All strains possessed genes associated with a broad spectrum of virulence such as toxin production, cell wall modifications, capsular polysaccharide, adhesion, proteolytic activity, and iron acquisition. Interestingly, eight strains were resistant to fosfomycin and four strains were resistant to erythromycin; also, all showed resistance to clindamycin. According to a review by Argemi et al., susceptibility to fosfomycin is greatly variable, with resistance reported in >50% of isolates. Moreover, the resistance to erythromycin and clindamycin is also emerging in such strains [26].

Together with antibiotic resistance, the biofilm formation is an important pathogenicity potential of the strain [28]. This feature is mainly important in infections associated with indwelling devices, but it is also known that the eradication of biofilm in chronic infections is of high priority [29]. In the case of our study, all *S. lugdunensis* strains were able to produce biofilm. Four strains produced biofilm at a high and very high level (OD_570_ values above 1.28). We depicted the scale of low, medium, and high biofilm formation based on two *S. aureus* strains, one with OD_570_ = 0.1446 as a negative control and one with OD_570_ = 1.2837 as a positive control. It should be noted that three strains produced biofilm at a rate that was two-fold higher than that of the positive control. Although all strains carried the *ica* locus, it is probably not the only cause of biofilm production. In staphylococci, several molecules other than polysaccharide intracellular adhesin (encoded by the *ica* operon) molecules have been shown to play an important role in biofilm formation, such as major autolysin Atl (*atlL*), the enzyme for D-alanine esterification of teichoic acids DltA (*dltA*), the iron-regulated surface determinant IsdC (*isdC*) under iron-limited conditions, and also the *agr* system, which is a component of the quorum-sensing *agr* system and is known to be a major element in the regulation of pathogenicity [30,31,32]. All tested *S. lugdunensis* strains possessed *atlL*, *dltA*, *isdC*, and *agr* genes.

As mentioned previously by Zipperer et al. [16] and later by Heilbronner and Foster in a review article [4], *S. lugdunensis* might be usable as a probiotic to reduce or eliminate *S. aureus* carriage when applied to nasal cavities. We decided to check if patients with chronic maxillary sinusitis were colonized with *S. aureus* at the time of *S. lugdunensis* isolation or before/after infection caused by *S. lugdunensis*. We found that eight patients had both *S. lugdunensis* and *S. aureus* in maxillary sinuses. Three of them before *S. lugdunensis* infection, one at the *S. lugdunensis* isolation, and five were infected with *S. aureus* after *S. lugdunensis* isolation.

Even though there are many open questions, it is speculated that *S. lugdunensis* is adapted to the presence of *S. aureus*. This may be explained by the fact that both species share the same niche but not the same adhesion sites. The success in nasal colonization by *S. aureus* is known to be multifactorial but mainly relies on cell wall adhesins such as ClfB or SdrD and wall teichoic acid molecules that interact with epithelial cells receptors [18]. The knowledge about the factors facilitating *S. lugdunensis* nasal colonization is insufficient. However, *S. lugdunensis* do not encode ClfB and SdrD, or a homologue of the *S. epidermidis* Aap protein, so it is not likely that the adhesion strategies are the same as in *S. aureus*. It is possible that *S. lugdunensis* uses *S. aureus* for its own benefit when the bacteria number is low and produces lugdunin to kill *S. aureus* when its number increases [4]. However, our findings are based on several strains; thus, it should be considered that *S. lugdunensis* and *S. aureus* may colonize the same niche, and further complex investigations are warranted.

## 4. Conclusions

In conclusion, within this study, we demonstrated several virulence factors carried by *S. lugdunensis* strains recovered from maxillary sinuses and associated with chronic disease. All tested strains were clustered in eight different STs. All tested strains were able to produce biofilm in vitro with numerous strains having a very strong ability. Overall, the strains were susceptible to most antibiotics tested in the study, but we found resistance to fosfomycin, erythromycin, and clindamycin in several strains. Moreover, the co-colonization of *S. lugdunensis* and *S. aureus* in the nasal cavity of laryngological patients should be reconsidered in the future. We believe that further in-depth analysis of *S. lugdunensis* strains from different niches, including the nasal one, should be performed in the future in order to reduce infection rates and broaden the knowledge about this opportunistic pathogen that is gaining attention.

## 5. Materials and Methods

### 5.1. Bacterial Isolates

The study included 18 *S. lugdunensis* clinical isolates recovered between 2017 and 2020 from laryngological patients treated in MML Medical Centre, Warsaw. The collection of bacterial isolates used in this study is described in Table 1. All isolates were recovered from maxillary sinuses. The PCM2430 (PL397 in this study) strain deposited in Polish Collection of Microorganisms (PCM) was used as a reference. All strains were cultivated on blood agar medium with 5% sheep blood (Graso Biotech, Starograd Gdański, Poland) in 37 °C for 20 h.

### 5.2. Genomic DNA Extraction

Genomic DNA extraction was performed as previously described [33]. Briefly, isolates were grown for 20 h at 37 °C on blood agar plates. A full inoculation loop of 10 μL of bacterial colonies was homogenized with a TissueLyser II (Qiagen, Germantown, MD, USA). The Qiagen DNeasy Blood & Tissue Kit (Qiagen, Germantown, MD, USA) was used for genomic DNA extraction. The subsequent steps were performed according to the manufacturer’s instructions. Purified DNA was stored at −20 °C.

### 5.3. Species Identification

All isolates were identified at the species level by sequencing the 16S rRNA and *rpoB* genes as previously described [34,35]. The PCR products were resolved by electrophoresis and purified using the Clean-Up Concentrator purification kit (A&A Biotechnology, Gdynia, Poland). Concentration and purity were measured using a NanoDrop ND-1000 (Thermo Fisher Scientific, Waltham, MA, USA). The PCR products were sequenced with the Sanger method at Genomed S.A. (Warsaw, Poland) with the same primers as those used for PCR.

### 5.4. Susceptibility Testing

Susceptibility testing was carried out according to the European Committee on Antimicrobial Susceptibility Testing (EUCAST; www.eucast.org/; Accessed on 11 February 2021) recommendations for all *S. lugdunensis* isolates. Disc diffusion values were performed for amikacin, cefazoline, cefoxitin, chloramphenicol, ciprofloxacin, clindamycin, erythromycin, gentamicin, linezolid, tetracycline, tigecycline, tobramycin, trimethoprim-sulfamethoxazole, rifampicin. Minimum inhibitory concentration (MIC) values for daptomycin, fosfomycin, teicoplanin, and vancomycin were determined using the Etest method.

### 5.5. Phenotypic Assessment of Secreted Virulence Factors Production

The secretion of virulence factors was evaluated using a culture-dependent assay with specific substratum for hemolysis and proteolysis detections [24]. All isolates were previously grown at 37 °C on TSA (Sigma-Aldrich, St. Louis, MO, USA). For hemolysin production, the *S. lugdunensis* isolates were cultivated on 5% sheep blood agar (Graso Biotech, Starograd Gdański, Poland), and the aspect of the hemolytic zone surrounding the colony was noted. For proteolysis, the isolates were cultivated onto TSA (Sigma-Aldrich, St. Louis, MO, USA) with 10% milk (0% fat).

### 5.6. Lugdunin Activity Assay

Lugdunin activity assay was performed as previously described [16], with modifications. Briefly, the antimicrobial activity was investigated against *S. aureus* USA300 ATCC BAA-1717. All *S. lugdunensis* strains and *S. aureus* USA300 were cultivated on TSA (Sigma-Aldrich, St. Louis, MO, USA) in 37 °C for 20 h. Then, a single colony was inoculated in 10 mL Basic Medium (BM: 1% tryptone, 0.5% yeast extract, 0.5% NaCl, 0.1% glucose and 0.1% K_2_HPO_4_, pH 7.2). Suspensions were incubated for 24 h at 37 °C with shaking (180 rpm). The *S. aureus* USA300 ATCC BAA-1717 strain was equated to OD_600_ = 0.5 (optical density measured at 600 nm wavelength) in fresh BM. In the next step, BM agar (BM: 1% tryptone, 0.5% yeast extract, 0.5% NaCl, 0.1% glucose and 0.1% K_2_HPO_4_, 1.5% agar, pH 7.2) supplemented with 200 μM 2,2′-bipyridine was used because, as previously described, isolates’ antimicrobial activity under iron-limiting conditions was higher [15]. So, BM agar plates were inoculated with an equated culture of *S. aureus* USA300 ATCC BAA-1717 in order to grow a bacterial lawn. All tested *S. lugdunensis* strains were inoculated in amount of 50 μL drop on the resulting *S. aureus* bacterial lawn, and the plates were incubated at 37 °C. After 24 h and 48 h incubation, the zone sizes were measured.

### 5.7. Biofilm Formation Assay

The quantitative assay for biofilm formation was carried out as previously described, with several modifications [36]. Briefly, all *S. lugdunensis* isolates were grown overnight at 37 °C as pure cultures on Tryptic Soy Agar (TSA) (Sigma-Aldrich, St. Louis, MO, USA). The single colony was inoculated in 10 mL Tryptic Soy Broth (TSB) (Sigma-Aldrich, St. Louis, MO, USA) and incubated for 24 h at 37 °C. After overnight culture, bacterial suspensions were equated to OD_600_ = 0.5 (optical density measured at 600 nm wavelength) with fresh TSB (Sigma-Aldrich, St. Louis, MO, USA). Then, suspensions were diluted 1:100 with fresh TSB (Sigma-Aldrich, St. Louis, MO, USA) with 1% glucose (POCH, Gliwice, Poland). For each isolate, 200 µL aliquots of prepared suspension were inoculated into four wells of the 96-well tissue culture plates (Biologix, Jinan, China). Each culture plate included an experiment negative control (TSB with 1% glucose) and biofilm negative activity control (*S. aureus*, CH21 strain). Ap *S. aureus* (LS1) strain that is a hyperproducer of biofilm formation was used as a positive control. The plates were incubated at 37 °C for 48 h. Afterwards, content of each well was removed by aspiration and the wells were rinsed three times with 300 µL sterile phosphate buffer solution (PBS) (Sigma-Aldrich, St. Louis, MO, USA). The plates were dried in 60 °C for 1 h and afterwards stained with 150 µL 0.5% crystal violet solution (Honeywell Fluka, Charlotte, NC, USA) for 15 min at room temperature. Following staining, the plates were rinsed using distilled water until there was no visible trace of stain. The stain bound to bacteria was dissolved by adding 150 µL of 33% acetic acid solution (POCH, Gliwice, Poland). The OD of each well was measured using a spectrometric reader (FlexiStation 3, Molecular Devices, San Jose, CA, USA) at 570 nm (OD_570_). Biofilm formation data were analyzed with the Bonferroni’s Multiple Comparison Test using GraphPad Prism 5 software (San Diego, CA, USA).

### 5.8. MLST Typing

The multilocus sequence typing (MLST) was carried out according to the protocol for *S. lugdunensis* on the MLST website (https://bigsdb.pasteur.fr/staphlugdunensis/; Accessed on 26 July 2021) and as previously described [25]. For *ddl* gene amplification, primers ddl-F-5′-CAAAATGTATTAAATGCTAT-3′ and ddl-R-5′-ATTTAATGATATTTCCTTGAT-3′ (product size 421 bp) were used. Briefly, all housekeeping genes were amplified with PCR and sequenced, then, alleles and sequence types (STs) for allelic profiles were assigned according to the *S. lugdunensis* MLST website.

### 5.9. Molecular Detection of Virulence Genes

The PCRs targeted to detect the presence of the following genes: the accessory gene regulator (*agr*) [37], the staphylococcal accessory regulator (*sarA*) [38]*,* adhesins genes (*atlL*) [39], haemolysins genes (*hlb, hem-III*) [40], the genes encoding proteins of the extracellular matrix (*fbl*, *vwbl*) [40,41], *S. lugdunensis* synergistic hemolysins (*slush*) [40] and proteolytic activity—lugdulysin (*shpI*) [42], were performed. The *agr* types were identified based on the expected product sizes (586 bp for *agr* type I and 771 bp for *agr* type II). Detection of lugdunin operon (*lugRDCA*), biofilm synthesis gene (*icaA*), adhesins genes encoding laminin and elastin binding protein (*eno*, *ebpS*), capsular polysaccharide biosynthesis protein gene (*capA*), cell walls modifications genes (*mprF* and *dltA*), and iron surface determinant genes (*isdC*, *sirA*, *lhaS*) was performed with sequencing primers designed in the current study (Table 2). The PCR products were resolved by electrophoresis, and the band patterns were analyzed.

### 5.10. Nucleotide Sequence Accession Numbers

The 169 sequences for one *Staphylococcus lugdunensis* were annotated using the NCBI BankIt tool and deposited in the GenBank database (https://www.ncbi.nlm.nih.gov/genbank/ accessed on 26 May 2022) under the following accession numbers: for the 16S rRNA gene, ON584774-ON584791; for the *rpoB* gene, ON604987-ON605004; for the *aroE*, *dat*, *ddl*, *gmk*, *ldh*, *recA* and *yqiL* genes, ON605005-ON605137, respectively.

### 5.11. Statistical Analysis

Biofilm formation experimental data were analyzed using ANOVA test followed by the Bonferroni’s multiple comparison test to evaluate the difference between groups. Before analysis, normality was checked with the Shapiro–Wilk test. All analyses were performed using GraphPad Prism (GraphPad Software, Inc., La Jolla, CA, USA; version 5.03) and data were presented as the mean ± standard deviation. *p* value < 0.05 was considered to indicate a statistically significant difference.

## Figures and Tables

**Table 1:** Characteristic of patients and isolates from chronic sinusitis.

			Clinical Characteristic of Patients					Microbiological Cultures—Characteristic	
Isolate Name	Date of Strain Isolation	Isolation Site	Age	Gender	Underlying Diseases	Additional Patient-Associated Risk Factors	Surgery	Accompanying Bacterial Flora at Isolation Time	Isolation of *S. aureus*
WAW2480	6 September 2017	left maxillary sinus	59	M	chronic sinusitis	none	FESS, DSN, HCNI	*S. epidermidis*; *S. salivarius*; *S. mitis*	no
WAW2487	8 September 2017	right maxillary sinus	36	M	chronic sinusitis	sleep apnea	DSN, HCNI, UPP	*E. coli*	no
WAW2660	31 October 2017	right maxillary sinus	56	M	chronic sinusitis	none	FESS, DSN	*S. epidermidis*	no
WAW2787	13 January 2018	right maxillary sinus	43	M	chronic sinusitis	sleep disturbance; snoring	DSN, HCNI	no data	no data
WAW2837	8 February 2018	left maxillary sinus	39	M	chronic sinusitis	nasal polyps; lower nasal concha hypertrophy	FESS	*S. epidermidis*; *Moraxella catarrhalis*; *C. pseudodiphtheriticum*	no
WAW2892	24 February 2018	right maxillary sinus	35	M	chronic sinusitis	none	FESS	*S. salivarius S. hominis*	no
WAW2923	8 March 2018	left maxillary sinus	64	M	chronic sinusitis	snoring	FESS, DSN, HCNI	no data	no data
WAW3029	13 April 2018	right maxillary sinus	46	M	chronic sinusitis	snoring	MIST	*S. epidermidis*	yes ^B^
WAW3032	13 April 2018	right maxillary sinus	31	M	chronic sinusitis	recurring respiratory infections; snoring	FESS, HCNI	*S. epidermidis*; *S. mitis*; *S. capitis*; *Pantoea agglomerans*	yes ^A,C^
WAW3253	13 June 2018	left maxillary sinus	44	M	chronic sinusitis	none	FESS, DSN, HCNI (twice), CELON	*S. pneumoniae*; *Propoonibacterium acnes*; *S. epidermidis*	yes ^C^
WAW3316	18 July 2018	left maxillary sinus	23	M	chronic sinusitis	none	Endoscopic sinus catheterization, DSN, HCNI	*S. epidermidis*; *C. accolens*	no
WAW12	2 April 2019	right maxillary sinus	36	M	chronic sinusitis	none	FESS, DSN, HCNI	*C. pseudodiphtheriticum*; *Klebsiella oxytoca*; *S. epidermidis*	no
WAW75	27 April 2019	left maxillary sinus	34	M	chronic sinusitis	none	FESS, DSN, HCNI	*S. epidermidis*; *E. cloacae*	yes ^C^
WAW120	20 May 2019	left maxillary sinus	43	M	chronic sinusitis	nasal polyps; lower nasal concha hypertrophy	FESS, DSN, HCNI	*Citrobacter freundii*; *S. epidermidis*	yes ^C^
WAW168	12 June 2019	right maxillary sinus	26	M	chronic sinusitis	nasal polyps; lower nasal concha hypertrophy	FESS, DSN, HCNI	-	no
WAW209	31 July 2019	right maxillary sinus	56	M	chronic sinusitis	nasal polyps	FESS, DSN, HCNI	*S. epidermidis*	no
WAW297	15 October 2019	left maxillary sinus	48	M	chronic sinusitis	nasal polyps	FESS, DSN, HCNI	-	no
WAW435	25 June 2020	right maxillary sinus	56	M	chronic sinusitis	nasal polyps; lower nasal concha hypertrophy; sleep apnea.	FESS	*S. epidermidis*	yes ^A^

^A^ before isolation; ^B^ at the isolation time; ^C^ after isolation. FESS—Functional Endoscopic Sinus Surgery. DSN—Deviated Septum Surgery. HCNI—Correction of lower nasal turbinates. MIST—Minimally invasive sinus technique. UPP—Uvulopalatopharyngoplasty.

**Table 2:** The nucleotide sequences of primers designed within this study and used for the detection of the virulence genes.

Virulence Factor(s)	Gene(s)	Primers	Sequence (5′–3′)	T_m_ (°C)	Product Size (bp)
**Lugdunin**	*lugR*	lugR_F	TGAAGTCATCATAAGTGCACACAA	50	296
		lugR_R	ATCCTAAGGCAGAAATCCCTAAAT		
	*lugD*	lugD_F	ACACAAGCGAAAGCGTTCAT	48	717
		lugD_R	GGCTACTCCCATTCCACCAA		
	*lugC*	lugC_F	AAACGCATTCTGGACGGGAT	50	994
		lugC_R	TTTGGGTTGCCCGTAGTACC		
	*lugA*	lugA_F	ACCACATAATTGCGAAGGCG	50	1396
		lugA_R	AGCCTCCATGTTTCCATGGTT		
**Biofilm synthesis**	*icaA*	icaA _F	ATGAAATATTTAAATTTGTTAA	43	1224
		icaA _R	CTAATTTTTTCCTCTGTCTGG		
**Adhesins/MSCRAMMs**	*eno*	enoF	AGCTACTGCGATGTCAGCAA	50	1059
		enoR	GCATTAGTGCCATCAGGTGC		
	*ebpS*	ebpSF	CGTCAGCGGAACACCAAAAG	50	969
		ebpSR	ATTTGACTGTGACGCTCCGT		
**Capsular polysaccharide biosynthesis protein**	*capA*	capAF	ATGGAAAAAACGCTTGATT	40	663
		capAR	CTATTTCAATTTATGGATT		
**Cell walls modifications**	*dltA*	dltAF	GACGTGCAACACCTACTGGA	50	925
		dltAR	GATATTGAGCAAGCGCAGCC		
	*mprF*	mprFF	TGCCACAACGACAGGTACAA	50	728
		mprFR	TCAATCGCTGGATGCTCGTT		
**Iron surface determinants**	*isdC*	isdCF	TCGCAGAGGGTCAGTCACTT	50	429
		isdCR	CACTTGCTGCTGAGCCTGTA		
	*sirA*	sirAF	ATGAATAAAGTTGTTAACATTAT	40	993
		sirAR	TTACTTTGATTGTTTATCA		
	*lhaS*	lhaSF	ACCTGCCATGATTGGCTTTT	50	410
		lhaSR	TGTAACCTAGCCATGCACCAA		

## Data Availability

The datasets generated for this study can be found in Genbank ON584774-ON584791; ON604987-ON605004; ON605005-ON605137.
